# Cost-effectiveness analysis of lenvatinib treatment for patients with unresectable hepatocellular carcinoma (uHCC) compared with sorafenib in Japan

**DOI:** 10.1007/s00535-019-01554-0

**Published:** 2019-02-20

**Authors:** Masahiro Kobayashi, Masatoshi Kudo, Namiki Izumi, Shuichi Kaneko, Mie Azuma, Ronda Copher, Genevieve Meier, Janice Pan, Mika Ishii, Shunya Ikeda

**Affiliations:** 1 Toranomon Hospital, Minato-ku, Tokyo Japan; 2 Kindai University, Osakasayama, Osaka Japan; 3 Musashino Red Cross Hospital, Musashino, Tokyo Japan; 4 Kanazawa University, Kanazawa, Ishikawa Japan; 5 Eisai Co., Ltd., Bunkyo-ku, Tokyo Japan; 6 Eisai Inc., Woodcliff Lake, NJ USA; 7 International University of Health and Welfare, Narita, Japan

**Keywords:** Lenvatinib, Sorafenib, Hepatocellular carcinoma, Cost-effectiveness, QALY

## Abstract

**Background:**

Lenvatinib demonstrated a treatment effect on overall survival by the statistical confirmation of non-inferiority to sorafenib for the first-line treatment of uHCC. The objective of this study was to evaluate the cost-effectiveness of lenvatinib compared with sorafenib for patients with uHCC in Japan.

**Methods:**

A partitioned-survival model was developed to estimate the cost-effectiveness of lenvatinib versus sorafenib when treating uHCC patients over a lifetime horizon and considering total public healthcare expenditure. Efficacy and safety data were extracted from the REFLECT trial. Utility values were derived from the European Quality-of-Life 5-Dimension Questionnaire, conducted with patients enrolled in the REFLECT trial. Direct medical costs, such as primary drug therapy, outpatient visits, diagnostic tests, hospitalization, post-progression therapy, and adverse-event treatments, were included. Cost parameters unavailable in the clinical trial or publications were obtained based on the consolidated clinical standards from a Delphi panel of four Japanese medical experts.

**Results:**

For lenvatinib versus sorafenib, the incremental cost was − 406,307 Japanese Yen (JPY), and the incremental life years and quality-adjusted life years (QALYs) were 0.27 and 0.23, respectively. Thus, lenvatinib dominated sorafenib, due to the mean incremental cost-effectiveness ratio falling in the fourth quadrant, conferring more benefit at lower costs compared with sorafenib. The probabilistic sensitivity analysis showed that 81.3% of the simulations were favorable to lenvatinib compared with sorafenib, with a payer’s willingness-to-pay-per-QALY of 5 million JPY.

**Conclusions:**

Lenvatinib was cost-effective compared with sorafenib for the first-line treatment of uHCC in Japan.

**Electronic supplementary material:**

The online version of this article (10.1007/s00535-019-01554-0) contains supplementary material, which is available to authorized users.

## Introduction

Hepatocellular carcinoma (HCC) is the most common type of liver cancer [1]. In Japan, approximately 40,000 patients are newly diagnosed with HCC each year; in 2018, there were 27,000 deaths estimated from HCC alone (https://ganjoho.jp/en/public/statistics/short_pred.html). HCC is strongly related to chronic hepatitis and cirrhosis derived from hepatitis B or C virus infection [1].

Japanese HCC patients are likely to have a hepatitis C infection, similar to their Western counterparts [2, 3]. The other risk factors include being male, older age, higher alcohol use, a higher alpha-fetoprotein (AFP) level, or a low platelet count [2, 4, 5]. A few subjective symptoms due to HCC may affect risk, such as anorexia, gastrointestinal symptoms, and jaundice.

There are limited treatment options for HCC patients who are ineligible for surgical resection. Locoregional therapies, such as radiofrequency ablation, transarterial chemoembolization (TACE), transarterial embolization (TAE), or hepatic arterial infusion chemotherapy (HAIC), are primarily recommended, and if one of those fail, then systemic therapy is considered. The 2013 Japan Society of Hepatology HCC Guidelines outlined that the factors influencing treatment decisions should be based on the degree of liver damage (Child–Pugh), presence or absence of extrahepatic spread and macrovascular invasion, the number of tumors, and tumor diameter [6].

Sorafenib, an oral multikinase inhibitor, has been the only systemic therapy demonstrated to extend OS as a first-line treatment, showing a median improvement of 2.8 months compared with placebo (10.7 months vs. 7.9 months; hazard ratio [HR] 0.69; *p* < 0.001) [7]. In patients from the Asia–Pacific region taking sorafenib, the median OS (mOS) improvement compared with placebo was 2.3 months (HR 0.68; *p* = 0.014) [8]. The use of other molecularly targeted agents has not demonstrated efficacy via non-inferiority [9–11] or superiority [12] to sorafenib; thus, until the appearance of lenvatinib, sorafenib has also been widely used as the first-line treatment for uHCC patients in Japan [13, 14]. Recently, regorafenib was approved as a second-line systemic treatment for patients who do not respond to the first-line treatments [15].

Lenvatinib is a multiple receptor tyrosine kinase inhibitor that acts on vascular endothelial growth factor receptors (VEGFR1-3), fibroblast growth factor receptors (FGFR1–4), platelet-derived growth factor receptor alpha (PDGFR-alpha), KIT, and RET to inhibit pathogenic angiogenesis. Lenvatinib monotherapy is approved for the treatment of radioiodine-refractory differentiated thyroid cancer [16] worldwide, including Japan. Lenvatinib and everolimus were approved as a combined treatment for advanced renal cell carcinoma following 1 previous antiangiogenic therapy in the United States, the European Union, and several other countries, but not in Japan [17]. More recently, lenvatinib was approved first in Japan (March 2018) as a first-line treatment for unresectable hepatocellular carcinoma (uHCC).

Lenvatinib was investigated for the treatment of uHCC in the phase 3 REFLECT (Study E7080-G000-304—Eisai) trial. REFLECT [A Multicenter, Randomized, Open-Label, Phase 3 Trial to Compare the Efficacy and Safety of Lenvatinib (E7080)Versus Sorafenib in the First-Line Treatment of Subjects With Unresectable Hepatocellular Carcinoma] was a non-inferiority trial conducted in 20 countries worldwide. A total of 954 eligible patients with confirmed uHCC were randomly assigned to receive oral lenvatinib (*n* = 478; 12 mg/day for a bodyweight of ≥ 60 kg or 8 mg/day for a bodyweight of < 60 kg) or sorafenib (*n* = 476) 400 mg twice daily in 28-day cycles. The primary end point was OS, with the non-inferiority margin set at 1.08. The lenvatinib median survival time of 13.6 months (95% confidence interval [CI] 12.1–14.9) was non-inferior to that of sorafenib (12.3 months, 10.4–13.9; HR 0.92, 95% CI 0.79–1.06). The median duration of study treatment for patients in the lenvatinib group was 5.7 months compared with 3.7 months in the sorafenib group. Relative to sorafenib, treatment with lenvatinib resulted in a statistically significant improvement in progression-free survival (PFS) of 7.4 months (95% CI 6.9–8.8), while sorafenib treatment PFS was 3.7 months (3.6–4.6; HR 0.66, 95% CI 0.57–0.77, *p* < 0.0001) [18]. Recently, there is an increasing awareness of about rising health care costs into innovative and expensive pharmaceuticals [19, 20]. The objective of this study was, therefore, to evaluate the cost-effectiveness of lenvatinib compared with sorafenib for the first-line treatment of uHCC patients in Japan, using patient-level data from the REFLECT trial and considering total Japanese public healthcare expenditure.

## Methods

### Model structure

We implemented a partitioned-survival model (PSM), using mature patient-level data from REFLECT. Trial-based Kaplan–Meier curves and cumulative survival probabilities were used to determine the proportion of patients in each health state. A PSM is commonly used in the late-stage oncology modeling (http://www.yhec.co.uk/glossary/partitioned-survival-model/). A PSM was developed in Microsoft^®^ Excel over a lifetime horizon.

As shown in the online Supplemental Figure 1, patients are in 1 of 3 distinct and mutually exclusive health states at the end of each 28-day model cycle:

- Progression-free: time from randomization to the first documentation of disease progression or death, whichever occurred first.
- Post-progression: time from the date of first documentation of disease progression until death using the modified response evaluation criteria in solid tumors (mRECIST).
- Death.

By incorporating these health states, the model captures the chronic and progressive nature of uHCC and reflects the key outcomes of the REFLECT trial. These are the 3 most relevant health states from a patient, clinician, and payer perspective, as 2 of the primary objectives of treating advanced HCC are prolonging life and avoiding disease progression [21].

### Patient treatment pathways

The REFLECT trial enrolled patients with intermediate and advanced stage uHCC (Barcelona Clinic Liver Cancer Stage “B” or “C”). The treatment pathway was based on the clinical trial and we assumed the pathway matched clinical practice in Japan. There are only 3 agents recommended as systemic therapy in Japan: sorafenib and lenvatinib for first-line (i.e., primary) systemic therapy and regorafenib for second-line systemic therapy. In this analysis, sorafenib was set as the study comparator, and regorafenib was used as an option for post-progression therapy, including TACE and HAIC.

### Clinical outcomes

#### Efficacy

REFLECT demographic characteristics were generally well balanced between the lenvatinib and sorafenib arms, but the proportion of patients with baseline AFP levels of ≥ 200 ng/mL, an adverse prognostic factor in HCC [2, 6], was greater in the lenvatinib arm than in the sorafenib arm: 46.4% versus 39.3%, respectively. Consequently, there were fewer patients with AFP levels < 200 ng/mL in the lenvatinib arm than the sorafenib arm: 53.3% and 60.1%, respectively. One of the most important prognostic factors for survival in HCC is elevated AFP levels. Indeed, AFP levels in adult patients with HCC are directly proportional to cancer size and tend to parallel the tumor-volume doubling time [22]. A plan to perform covariate analyses for supporting the efficacy results was included in the statistical analysis plan of the REFLECT trial. Kudo et al. (see supplementary appendix Table S2 of [18]) [18] reported adjusted HRs (based on a Cox regression model including treatment group and the respective baseline characteristics), where stratifying for AFP was the only variable to produce a nominally significant OS HR (0.856, CI 0.736–0.995). In consideration of this, the cost-effectiveness analysis used clinical outcomes from the AFP-adjusted population of the REFLECT trial in the base case. The intent-to-treat (ITT) population from the REFLECT trial was included as a scenario analysis in the results.

In the REFLECT trial, 73.4% and 73.5% of patients in the lenvatinib and sorafenib trial arms, respectively, died and 64.4% and 72.1% had experienced disease progression, respectively; thus, extrapolating PFS and OS data beyond the end of the REFLECT trial was needed. Extrapolation was achieved using parametric survival analysis, performed in accordance with the relevant National Institute for Health and Clinical Excellence Decision Support Unit guidance [23]. Parametric survival models were generated with AFP adjustment for 6 parametric distributions, namely Weibull, exponential, log-logistic, log-normal, generalized gamma, and Gompertz. The analysis adjusting for AFP used the mean of covariates method. The most appropriate distribution (of the 6) was selected based on:

- Assessment of the statistical goodness of fit (measured using the Akaike Information Criterion [AIC] and Bayesian Information Criteria [BIC])
- Consistency with the previous findings of extrapolation methods in advanced HCC.

Model fit statistics (AIC/BIC) for the lenvatinib and sorafenib data suggested that the log-logistic distribution was preferred for OS. For PFS, the statistical measures indicated that the log-normal was preferred for lenvatinib, while the gamma distribution was preferred for sorafenib. However, use of the gamma distribution led to implausible extrapolations in which PFS for sorafenib exceeded that of lenvatinib. This was not considered a clinically plausible scenario. The log-normal distribution was the next-best-fitting distribution and provided clinically plausible results and was, therefore, used in the base case. Thus, these distributions were applied in the base case.

#### Adverse events

We included AEs that had an impact on a patient’s quality of life (QoL). We excluded asymptomatic AEs, such as decreased platelet count and increased aspartate aminotransferase, which only affected biological or immunological markers, but that had no impact on the patient’s QoL. Rates of grade 3 or 4 treatment-emergent AEs, along with the Common Terminology Criteria, occurring in at least 1% of subjects in either treatment arm, came from the REFLECT trial. In the lenvatinib and sorafenib arms, AEs included: diarrhea (4% vs 4%), asthenic conditions (3% vs 2%), decreased appetite (5% vs 1%), abdominal pain (2% vs 3%), decreased weight (8% vs 3%), vomiting (1% vs 1%), hypertension (23% vs 14%), and palmar–plantar erythrodysesthesia/hand–foot syndrome (3% vs 11%), respectively [18].

### Utilities

Utility values in the model were derived through analysis of the patient-level data in the REFLECT trial, using the 3-level version of the European Quality-of-Life 5-Dimension Questionnaire (EQ-5D-3L). Patients completed an EQ-5D-3L at the baseline visit, on day 1 of each cycle after cycle 1, and at the off-treatment visit, which occurred within 30 days after the final administration of the study drug.

The baseline utility for a progression-free patient without drug treatment was 0.845. The utility values for a patient with progression were 0.714 in 2 treatment arms. For patients who were progression-free with treatment, the utility is calculated by the sum of progression-free disease without treatment utility and the sum of AE disutilities for each treatment, resulting in 0.832 for Lenvatinib and 0.837 for Sorafenib. Each AE disutility is calculated by multiplying the AE prevalence rate by a given disutility. The sum of all AE disutilities was 1.253 × 10^−2^ for lenvatinib and 0.809 × 10^−2^ for sorafenib, reported by Hudgens et al. reported at ISPOR Europe 2017 and ISPOR US 2018. Though not measured by the EQ-5D-3L instrument in the REFLECT trial, hypertension is a relevant AE. To account for this, we used a zero value for disutility in the base case (Tables 1, 2).

**Table 1:** Disutility related to grade 3–4 adverse events

	Disutility values	Disutility calculation	
		Lenvatinib (× 10^−2^)	Sorafenib (× 10^−2^)
Diarrhea	− 0.014	− 0.059	− 0.059
Asthenic conditions	− 0.108	− 0.318	− 0.250
Decreased appetite	− 0.078	− 0.361	− 0.099
Abdominal pain	− 0.005	− 0.008	− 0.014
Weight decreased	− 0.053	− 0.401	− 0.156
Vomiting	− 0.047	− 0.059	− 0.049
Hypertension	0.000	0.000	0.000
Hand–foot syndrome/PPES	− 0.016	− 0.047	− 0.182
Total		− 1.253	− 0.809

*PPES* palmar–plantar erythrodysesthesia syndrome

**Table 2:** Health-state utility values including adverse events

	Lenvatinib	Sorafenib
Progression-free disease state without treatment (baseline)	0.845	0.845
Progression-free disease state with treatment	0.832	0.837
Post-progression state	0.714	0.714

### Healthcare resource use

As there are limited available data for the remaining healthcare resource use, we conducted a Delphi panel with 4 Japanese medical experts to consolidate resource utilization information for uHCC standard treatment and treatment pathways.

The Delphi method is a structured communication technique, using surveys and subsequent rounds of feedback to generate expert consensus [24]. It comprised sequential questionnaires answered anonymously by a panel of participants with relevant HCC expertise. After each questionnaire, a summary of the group’s responses was fed back to the participants. To consolidate data from the experts, we conducted a paper survey as a first round, and then held a face-to-face meeting afterwards, as part of a modified Delphi process.

Experts were queried on the medical resource use of uHCC patients in progression-free and post-progression health states, such as the number of physician visits, laboratory and radiological tests, proportion of patients requiring hospital admission, number of hospitalizations, number of days per hospital admission stay, and types and frequency of monitoring tests. Delphi panelists were also questioned on the proportion of patients moving to post-progression therapy, the types of post-progression treatments, and the proportion of each treatment that patients received in the clinical practice. Because lenvatinib was not approved for HCC treatment in Japan when we initiated the analysis, we obtained the medical resource use for sorafenib treatment in practice and assumed the same medical resource use for lenvatinib.

We defined hospitalization broadly as when treatment required for unexpected conditions including adverse events in the Delphi study, because there is large variation between institutions regarding the definition of hospitalization of patients.

We considered that best supportive care (BSC) or palliative care was given to any patient on any treatment, including those not receiving treatment. Because there were no studies demonstrating BSC costs in Japan, we set BSC and palliative care costs to zero in this study.

Finally, the model included mortality as costs associated with end-of-life (EoL) medical resource use.

### Healthcare costs

We included direct healthcare costs, namely: (1) primary drug-therapy costs, which included drugs and drug administration; (2) medical resource-use costs, which included outpatient visits, laboratory tests radiological tests, and hospitalization; (3) AE treatment costs; (4) post-progression therapy costs; and (5) EoL costs. Indirect costs, such as caregiver costs or productivity losses arising from an inability to work, were excluded. All costs were reported in Japanese Yen (JPY) 2017 value (Table 3).

**Table 3:** Direct healthcare costs

Categories		Costs per cycle (JPY)
Primary drug-therapy costs		
Lenvatinib^a^		263,861.76
Sorafenib		436,180.30
Medical resource costs		
Progression-free		47,312.33
	Outpatient visits	2056.06
	Laboratory tests^b^	20,093.91
	Radiological tests^c^	16,035.34
	Hospitalizations	9127.02
Post-progression		26,097.85
	Outpatient visits	759.98
	Laboratory tests^b^	10,423.59
	Radiological tests^c^	8505.24
	Hospitalizations	6409.04
Post-progression treatment costs		
Regorafenib		425,236.80
HAIC		91,745.29
TACE		84,597.08

Adverse event	Treatments	Costs per cycle (JPY)
Adverse-event treatment costs		
Diarrhea	Loperamide and bifidobacterium prescribed regularly	3147.20
Asthenic conditions	No treatment	N/A
Decreased appetite	No treatment	N/A
Abdominal pain	No treatment	N/A
Weight decreased	No treatment	N/A
Nausea	Domperidone prescribed regularly	1646.40
Rash symptoms	Diflorasone diacetate, heparinoid, and urea (all external applications) prescribed regularly	2353.00
Vomiting	Domperidone prescribed regularly	1646.40
Stomatitis	Triamcinolone (external application), prescribed as required, and vitamin-B-complex prescribed regularly	1092.00
Hypertension	Candesartan and amlodipine prescribed regularly	4900.00
Hand-foot syndrome/PPES	Diflorasone diacetate, heparinoid, and urea (all external application) prescribed regularly	1176.50

*AFP* alpha-fetoprotein, *HAIC* hepatic arterial infusion chemotherapy, *INR* prothrombin time and international normalized ratio, *PIVKA*-*2* protein induced by vitamin K absence-II, *PPES* palmar-plantar erythrodysesthesia syndrome, *TACE* transarterial chemoembolization

^a^Weighted total drug cost of 12 mg/day and 8 mg/day

^b^Includes AFP test, PIVKA-2, AFP-L3, INR, complete blood count, biochemistry, and endoscopy

^c^Includes CT scan, MRI scan, and ultrasounds

#### Primary drug-therapy costs

We used the reimbursement price of 3956.40 JPY for lenvatinib (per 4-mg capsule) and 4677.10 JPY for sorafenib (per 200-mg tablet), respectively, from the Japanese National Health Insurance (NHI) Drug Price List in 2017. Oral lenvatinib and oral sorafenib were prescribed in the outpatient setting and self-administered by patients in continuous 28-day cycles. Dose interruption, dose reduction, or treatment discontinuation was allowed for patients who experienced drug-related toxicity.

Primary drug-therapy costs per day were calculated as the number of pills per day multiplied by dose intensity and the NHI list price per pill. The dose intensity was calculated as the ratio of the actual dose to the planned starting dose. Dose intensity and body weight (as lenvatinib dose varies based on body weight) were extracted from patient demographics and clinical information from the REFLECT trial. Of the 954 ITT patients enrolled in the REFLECT trial, 478 were in the lenvatinib arm (of whom 68% were prescribed 12 mg/day). The mean dose intensity was 10.5 mg among patients treated with 12 mg/day (i.e., 88% of the planned starting dose), and 7.0 mg for those treated with 8 mg/day (correspondingly, also 88%). The mean sorafenib dose intensity was 663.8 mg, or 83% of the planned starting dose.

The total primary drug-therapy cost per cycle included an administrative cost for oral drugs based on the Japanese NHI Reimbursement Schedule.

#### Adverse-event treatment costs

Adverse-event treatment costs were included in the analysis for grade 3 or 4 treatment-emergent AEs occurring in at least 1% of subjects in either treatment arm. We considered that treatments were given to all patients with grade 3–4 AEs. Dosage and frequencies of the drug treatments were reviewed and confirmed by medical experts based on the treatments given in the clinical trial. We assumed that branded medicines were given to patients with AEs for the duration of treatment. Adverse-event costs were calculated using the NHI price of branded medicines for all AE treatments.

#### Medical resource-use costs

Medical costs were calculated as a fee-for-service system. Unit costs for outpatient visits, laboratory tests, radiological tests, and hospitalization were charged based on the NHI Reimbursement Schedule (Table 3).

#### Post-progression therapy costs

According to the Delphi panel, 52% of patients, after receiving sorafenib as primary drug treatment, moved to “no treatment,” while the remaining (48%) received 1 of the post-progression treatments of either regorafenib, HAIC, or TACE for 155 days (5.5 cycles). The Delphi process obtained the breakdown proportions of the post-progression treatments as regorafenib (32.2%), HAIC (23.7%), and TACE (44.1%) (Table 3).

#### EoL costs

The EoL costs for liver cancer patients were reported as 1 410 260 JPY in the last 3 months of life from a recent database analysis (reported by Hagino and Moriwaki at Annual meeting of Pharmaceutical Society of Japan 2018).

## Analyses

Costs and outcomes were discounted at 2% per year [25] over a time horizon of 65 years (849 cycles). We reported economic end points, namely quality-adjusted life years (QALYs) and life years (LYs), as well as incremental cost-effectiveness ratios (ICERs).

We carried out a deterministic sensitivity analysis (DSA) and probabilistic sensitivity analysis (PSA) to explore the potential influences of the different parameters in our analysis. In DSA, costs were varied ± 20% and dose intensities by ± 5%. Utilities, survival outcomes (OS, PFS), and treatment duration were varied based on ± 1.96 times their standard errors (SEs). The results are presented as a tornado diagram that shows the impact of the parameter variation on the model base-case ICER. For each PSA, 1000 simulations were drawn from the variables’ distributions. Costs were varied using a gamma distribution [26]. Utilities were assumed to follow a beta distribution [27]; a log-normal distribution was applied to PFS, while a log-logistic distribution was applied to OS [26]; and a normal distribution was assumed for dose intensity. The PSA was presented with the help of the probabilistic mean and SE. Results were also shown on a cost-effectiveness plane and in the form of cost-effectiveness acceptability curves (CEACs).

The Central Social Insurance Medical Council, Chu-i-kyo, has applied a cost-effectiveness threshold of 5 million JPY/QALY and 10 million JPY/QALY to the assigned 7 drugs for recalculation of list prices based on the tentative introduction of the cost-effectiveness assessment between 2016 and 2017 [28, 29]. We explored the conservative cost-effectiveness threshold of 5 million JPY/QALY in this analysis.

In addition to DSA and PSA, we conducted the following scenario analyses:

- *OS/PFS*—*ITT without stratification variables* In the base case, we used an AFP model with adjustments to OS/PFS. In the scenario analysis, we excluded this adjustment and assessed the impact of outcomes without any stratification.
- *Time horizon of 5 and 10* *years* PSM used extrapolated survival curves estimated from the phase 3 REFLECT trial data. As there are uncertainties around these extrapolated data, we reduced their impact by considering 2 scenarios with shorter time horizons.
- *Discount rate for costs and benefits set to 4%* Per Japanese guidelines, [25] we explored the alternative where the base-case discount rate for both costs and benefits was doubled.
- *Dose intensity and distribution aligned to the Japanese population* As Japanese patients generally have lower body weights than Western patients, we aligned these statistics to those of the Japanese (only) patients in the REFLECT trial, where: dose intensity of lenvatinib 8 mg = 80%, lenvatinib 12 mg = 70%, and sorafenib = 70%. Moreover, the distribution of patients treated with lenvatinib 8 mg versus 12 mg was assumed to be 50%.
- *The AE treatment cost was reduced by 60% of the base*-*case values* We reduced AE drug costs by 60% to account for generic drug prices.
- *Hypertension disutility from a systematic literature review* In the base case, we assumed a disutility of zero, due to the lack of appropriate literature. In the scenario analysis, we assumed a disutility of 0.012 for hypertension, which is the value of disutility-related AEs in the systematic literature review, although not specific to hypertension [30]. In an alternative scenario, we assumed a disutility of 0.028 for hypertension, which is the value of disutility from “any AE” from grade 1 to 4, extracted from the REFLECT trial.

## Results

### Base-case analysis (AFP covariate analysis)

In the base-case analysis, the mean estimated LYs and QALYs were 1.88 and 1.46 for lenvatinib treatment and 1.62 and 1.23 for sorafenib treatment, respectively (Table 4). The incremental LYs and QALYs were 0.27 and 0.23, respectively, for lenvatinib compared with sorafenib. Total costs of lenvatinib therapy were estimated at 5 088 957 JPY ($47 938 United States dollars [USD])^1^, while sorafenib costs were 5,495,264 JPY ($51,765 USD). The incremental cost was − 406,307 JPY ($3827 USD) for lenvatinib compared with sorafenib. Moreover, when lenvatinib is used instead of sorafenib, there was an estimated decrease in costs of 406,307 JPY ($3827 USD). Lenvatinib treatment is dominant versus sorafenib treatment, due to the mean ICER falling in the fourth quadrant, i.e., lenvatinib treatment conferred additional LY or QALY gains while reducing costs (Table 4).

**Table 4:** Results of the cost-effectiveness analysis (base-case analysis)

Treatment	Cost (JPY)	Effectiveness (LY)	Effectiveness (QALY)
Lenvatinib	5,088,957	1.88	1.46
Sorafenib	5,495,264	1.62	1.23
Incremental values	− 406,307	0.27	0.23
ICER		Dominant	Dominant

*ICER* incremental cost-effectiveness ratio, *LY* life year, *QALY* quality-adjusted life year

**Fig. 1:**
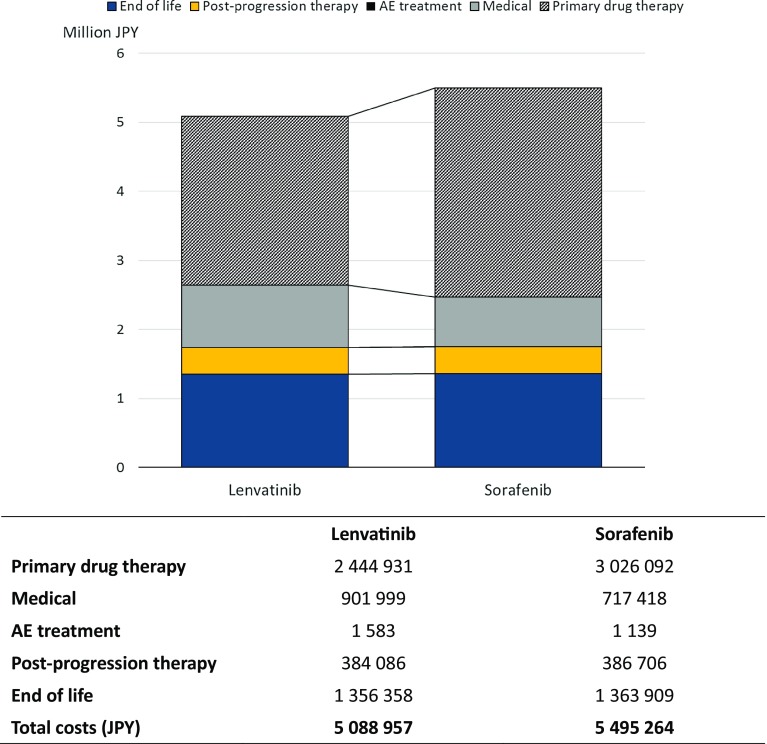
Breakdown of costs in the base case

In the breakdown of costs in Fig. 1, the cost structure was similar in both groups. It reveals that primary drug-therapy costs were the largest component of total costs, which accounted for approximately half of the total costs in both arms. The second largest cost component was EoL costs, followed by medical resource use, post-progression therapy, and AE treatment costs. Primary drug-therapy costs were 2.4 M JPY for lenvatinib and 3.0 M JPY for sorafenib. While the per-cycle drug cost of sorafenib is approximately 1.65 times higher than lenvatinib, significantly longer PFS and the longer treatment duration of patients on lenvatinib partially offset this cost, such that, over a lifetime horizon, the difference in primary drug-therapy cost of sorafenib falls to 1.24 times that of lenvatinib.

The difference in EoL costs between both therapies was small. This aligns with the clinical trial data (see Figure 2 of Kudo et al. [18]), where the OS was longer for lenvatinib, but both therapies had survival outcomes that were not statistically significantly different (HR 0.92, CI 0.79–1.06).

The medical resource-use costs associated with lenvatinib were approximately 20% higher than for sorafenib. This reflects, in part, the higher costs incurred by lenvatinib due to its significantly better PFS outcomes (see Figure 2 of Kudo et al. [18]). Medical resource-use costs are dependent on the post-progression medical costs incurred, which, for the same reason as above (i.e., longer survival), are likely to be higher for lenvatinib than sorafenib. Post-progression costs are determined beyond the clinical trial period and, hence, influenced by the methods used to extrapolate data. We discuss this further in the scenario analyses, in the following.

The AE treatment costs in both therapies were very small and accounted for less than 1% of the total cost, because no treatment was given to nearly half of the AEs. Although it was a small proportion, AE treatment costs associated with lenvatinib were slightly higher than with sorafenib because the treatment cost for hypertension, which occurred more frequently in lenvatinib than sorafenib, was the most expensive among those AE treatment costs per cycle.

### Sensitivity analysis

**Fig. 2:**
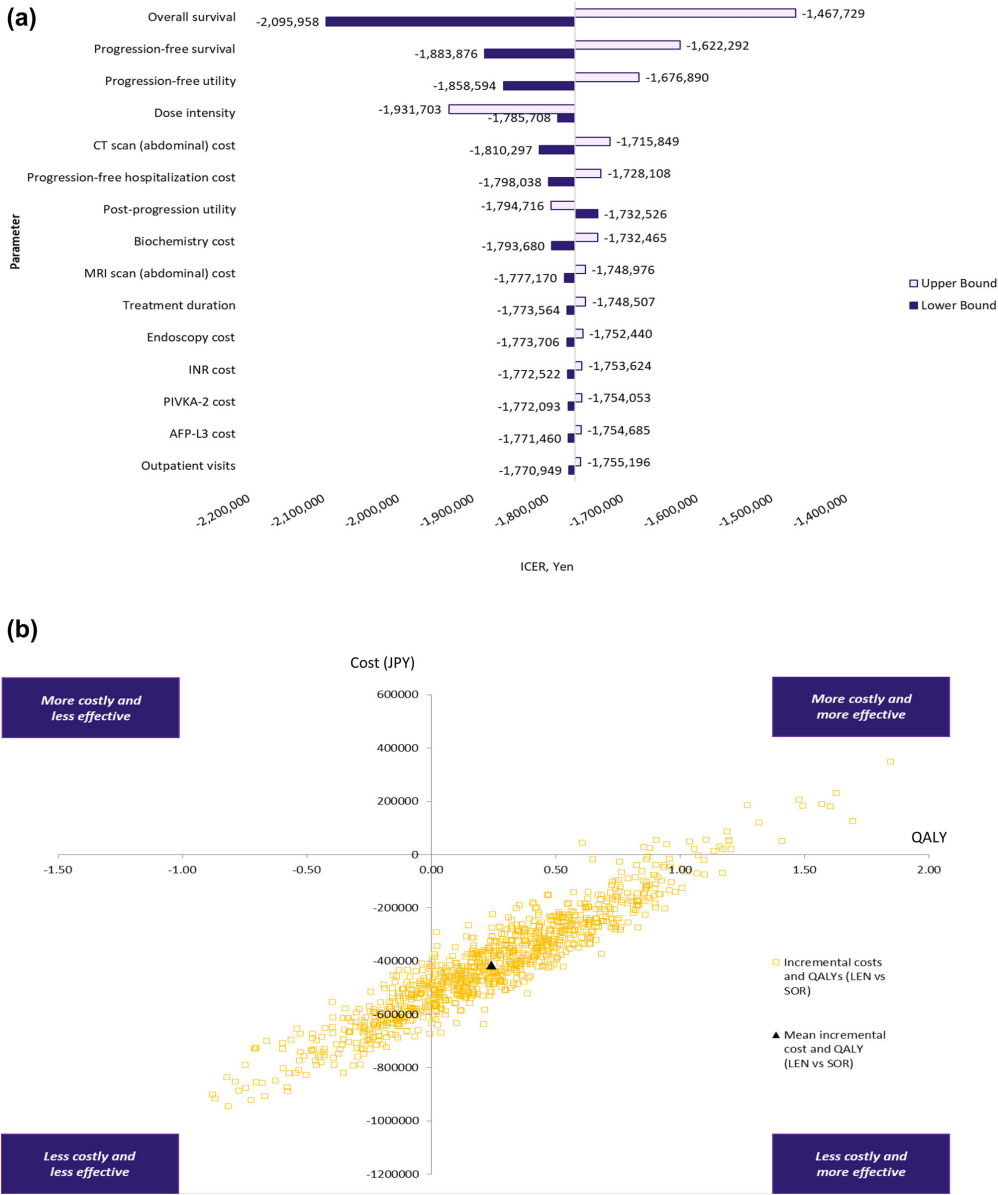

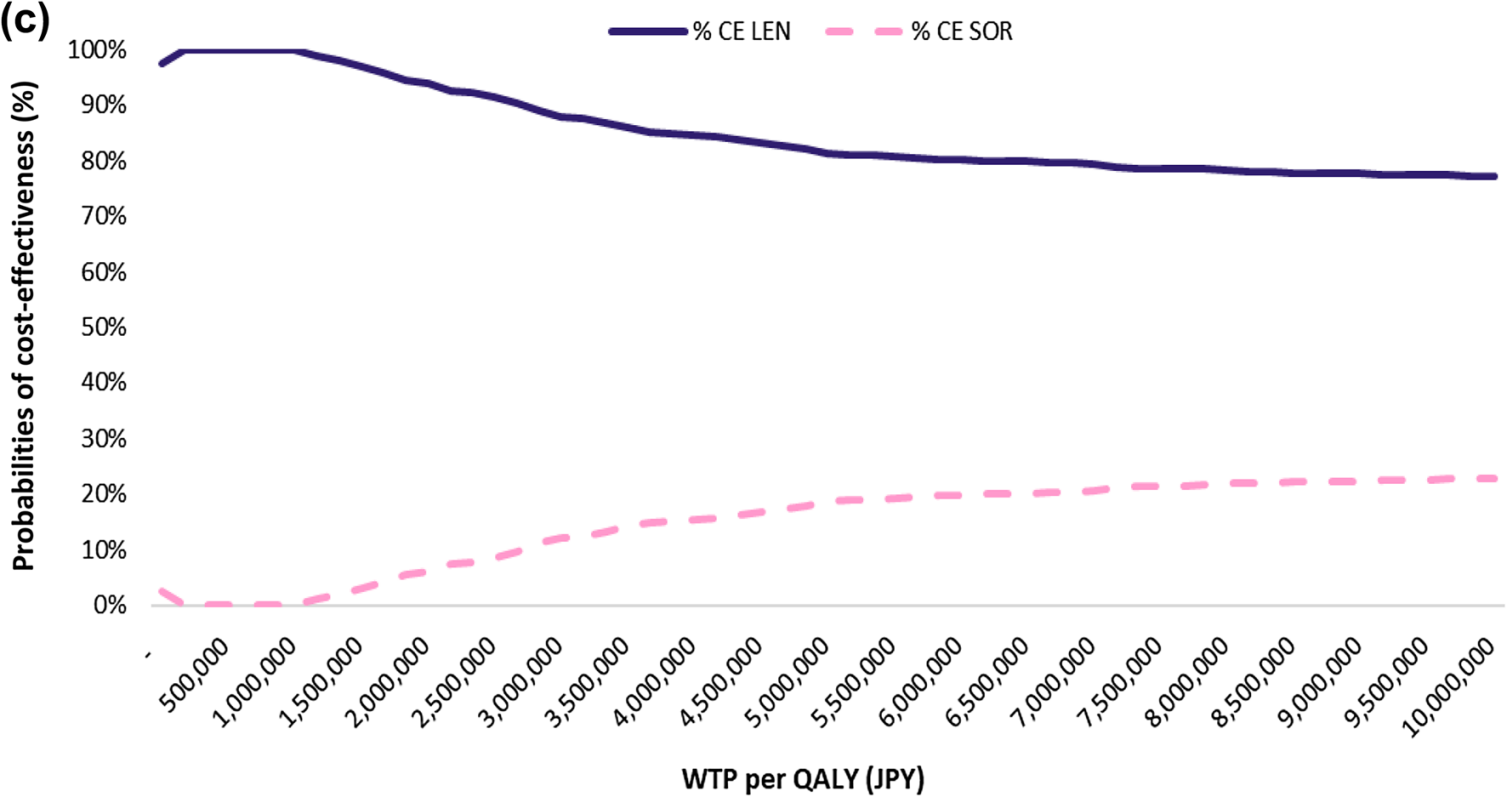
Deterministic sensitivity analysis (**a**), probabilistic sensitivity analysis (**b**), and cost-effectiveness acceptability curves (**c**), in lenvatinib compared to sorafenib. In a PSA, using a set of input parameter values drawn 1000 times by random sampling from each distribution, the model generates 1000 outcomes appeared as a “cloud” of potential outcomes (**b**), or graphed as a proportion in CEAC (**c**). **b** Visibly shown the four quadrants that the outcomes (i.e., ICER) can be fallen. The first quadrant is the area that lenvatinib is more effective (measured by QALY) and more costly compared with sorafenib. Similarly, the fourth quadrant is the area that lenvatinib is more effective and less costly compared with sorafenib. **c** tells that 81.3% of ICERs simulations fall the area considered cost-effective in relation to a given Japanese cost-effectiveness threshold of 5 million JPY per QALY. *AFP* alpha-fetoprotein, *CT scan* computed-tomography scan, *ICER* incremental cost-effectiveness ratio, *INR* prothrombin time and international normalized ratio, *LEN* lenvatinib, *MRI* magnetic resonance imaging, *PIVKA*-2 protein induced by vitamin K absence-II, *QALY* quality-adjusted life year, *SOR* sorafenib, *WTP* willingness-to-pay

A DSA was performed to assess the impact of the model parameter uncertainty on the base-case ICER (using QALYs as the outcomes measure). Figure 2 shows the results of the analysis. The 3 most significant model parameters were OS curves, PFS curves, and progression-free utility. Varying these 3 parameters still resulted in lenvatinib being dominant over sorafenib.

A PSA was performed to assess the impact of uncertainty on the model parameters. Figure 2 shows the cost-effectiveness plane and the CEAC for the PSA simulation. The CEAC shows the percentage of simulations favoring a treatment option. The CEAC shows that 81.3% of the simulations were favorable to lenvatinib compared with sorafenib when the payer’s WTP per QALY was 5.0 million JPY.

### Scenario analyses

The results of scenario analyses are shown in Table 5 and Supplemental Table 1. We show the base-case results in the top row as a basis for comparison. Lenvatinib remains dominant versus sorafenib across all scenarios. These findings confirm the robustness of our base-case findings.

**Table 5:** Scenario analyses results (lenvatinib vs sorafenib)

Description of scenario	∆ QALYs	∆ Costs^a^	ICER
*Base case*	*0.230*	− *406,307*	*Dominant*
OS/PFS—ITT without stratification variables	0.148	− 444,145	Dominant
Time horizon—5 years (65 cycles)	0.166	− 466,560	Dominant
Time horizon—10 years (130 cycles)	0.201	− 427,448	Dominant
Discount rate for costs and benefits: 4%	0.210	− 423,928	Dominant
Dose intensity and distribution^b^	0.230	− 479,438	Dominant
AE treatment cost: 60% of base case	0.230	− 406,485	Dominant
HTN disutility from SLR (= 0.012)	0.229	− 406,307	Dominant
HTN disutility from 304 (= 0.028)	0.228	− 406,307	Dominant

The “base case” results were already presented in the Table 4. However, for reference, to compare with the result of the scenario analysis, the base case results (in italic) were presented

*AE* adverse event, *HTN* hypertension, *ICER* incremental cost-effectiveness ratio, *ITT* intention-to-treat, *LY* life year, *OS* overall survival, *PFS* progression-free survival, *QALY* quality-adjusted life year

^a^Incremental costs (i.e., ∆) are reported in Japanese JPY 2017 value

^b^The dose intensity and distribution were aligned to the Japanese population

## Discussion

The cost-effectiveness analysis results showed lenvatinib treatment dominated sorafenib, due to the mean ICER falling in the fourth quadrant, as lenvatinib was more effective (measured by QALYs) and less costly compared with sorafenib for uHCC treatment in Japan. The significantly better PFS outcomes [18] resulted in higher QALYs for lenvatinib compared with sorafenib, because more time was spent in a better health state (i.e., progression-free). Moreover, using extrapolated data, lenvatinib patients remained in the progression-free health state as well as in the lifetime horizon longer than sorafenib patients, which also contributed to higher cumulative lifetime QALYs.

Another beneficial aspect of lenvatinib treatment was the cost-saving led by lower primary drug-therapy costs. This was despite more medical resource-use costs for lenvatinib. Due to oral administration, drug administration costs were the same for the 2 treatments, and thus, the major cost-saving driver was the drug cost. Dose intensity and body weight were extracted from patient demographics and clinical information from the REFLECT trial. We used the lenvatinib and sorafenib list prices of 3956.40 JPY (4-mg capsule) and 4677.10 JPY (200-mg tablet), respectively, from the Japanese NHI 2017 list of drugs. In Japan, the price of a new drug is determined based on its efficacy relative to “similar” comparators and is given the same daily costs as its comparator. In the case of lenvatinib, the price was based on sorafenib when lenvatinib was newly launched in the Japanese market in 2015. The daily dose of lenvatinib in differentiated thyroid cancer is twice the dose for HCC, whereas the daily dose of sorafenib for both HCC and differentiated thyroid cancer is identical. As a result, the total lenvatinib drug costs are nearly half sorafenib’s in HCC. Drug prices are revised every 2 years in Japan; the latest revision was in April 2018. The revised prices (as of 5 March 2018), where lenvatinib and sorafenib prices remain equivalent, are reflected in our analysis.

There are several limitations and data gaps in this analysis. First, we implemented a model analysis, using patient-level data from REFLECT trial. This may not necessarily reflect the real clinical efficacy and safety in the real world practice, while delivering such cost-effectiveness information at the timing when lenvatinib just launched would also be useful for physicians as well as for healthcare policy makers in terms of decision-making. To address such concern and to assess the robustness of our base-case analysis, we conducted a DSA and PSA to represent uncertainty by reporting the impact on cost-effectiveness following the standards of economic evaluation [31]. In the DSA, model parameters were varied over plausible values, with ICER varying between − 2,095,958 JPY (OS) and − 1,467,729 JPY (OS). The DSA results consistently support lenvatinib dominance versus sorafenib. In the PSA, simulation results were predominantly in the southeast quadrant of the cost-effectiveness plane (i.e., dominance). The CEAC showed that 81.3% of the simulations were favorable to lenvatinib compared with sorafenib when the payer’s WTP per QALY was 5.0 million JPY.

In addition, the cost-effectiveness analysis results were robust in the scenario analyses, which included: the ITT population from the REFLECT trial, an extrapolation without the AFP covariate stratification, shortening the model time horizon, doubling the discount rates, lowering AE treatment costs, larger hypertension disutility, and dose intensity and distribution aligned with Japanese population characteristics. Importantly, the model used extrapolated survival curves estimated from the REFLECT trial in uHCC patients; the median follow-up times, in months, were 27.7 for the lenvatinib treatment arm and 27.2 for the sorafenib treatment arm. Parametric survival model methods allowed us to extrapolate the data beyond the observed study period, but there are uncertainties associated with the extrapolation of survival curves beyond the observed data period. In the base case, the most appropriate distribution was selected using the following process: (a) assessment of the visual fit to the observed Kaplan–Meier, (b) assessment of the statistical goodness of fit (measured using the AIC and BIC), and (c) assessment of the plausibility of the long-term extrapolation. To address the uncertainty around the statistical methods used in extrapolating data, we explored alternative assumptions by varying the estimation models and functional forms for estimating OS/PFS in scenario analyses, and in all scenarios, our conclusions remain robust, namely that lenvatinib dominated sorafenib.

Second, we assumed that post-progression treatment after the first-line treatment with lenvatinib (when it is approved) would be the same as sorafenib. This was followed by the indication of regorafenib that the Ministry of Health, Labour and Welfare has granted regorafenib for the second-line treatment of patients with uHCC who have progressed after treatment with cancer chemotherapy. This was also consistent with 2018 practice guide by AASLD that the sequential use of multikinase tyrosine kinase inhibitors with similar mechanisms of action may be considered, although there are no specific data to support the use of regorafenib after progression on lenvatinib [32]. Further research may be necessary in the real world. On the premise of this, post-progression therapies used and the proportion of patients taking each therapy were obtained from medical experts through a Delphi panel. A Delphi panel was also necessary for 2 reasons: (1) the details of Japanese-specific data of the post-study therapies in the REFLECT trial were not available; (2) the post-study therapies given in the clinical trial for the ITT patients were different between the 2 arms due to the trial conditions. We assumed that the potential effect on OS from the Delphi-selected post-progression therapies was the same as those in REFLECT.

Third, utility values of both progression-free and post-progression health states were derived from EQ-5D-3L data in the REFLECT trial. As the post-progression utility data were obtained within the first month of entering the post-progression health state, the values are likely higher than if collected beyond the first month of post-progression or when a patient is closer to death. As such, it is likely that the utility values of the post-progression state in both groups of patients were overestimated, but a systematic literature review conducted in November 2017 was unsuccessful in finding alternative values from the existing literature in Japan.

Fourth, there is no consensus on the official WTP per QALY threshold in Japan. We used the conservative threshold of 5 million JPY per QALY. We found that lenvatinib is dominant versus sorafenib, suggesting that the threshold is not a constraint. However, in many cost-effectiveness analyses, the threshold is important for decision-making. The Chu-i-kyo is scheduled to discuss introducing a full-scale cost-effectiveness assessment in Japan; a decision may be reached by the end of the fiscal year 2018, including the WTP threshold that would apply in Japan.

## Conclusions

A cost-effectiveness analysis demonstrated that lenvatinib conferred more benefit at lower costs: it dominated sorafenib for the treatment of uHCC in Japan, due to the mean ICER falling in the fourth quadrant. The significantly better PFS outcomes observed in the trial resulted in higher QALYs for lenvatinib compared with sorafenib, because more time was spent in the progression-free health state. Moreover, using extrapolated data, lenvatinib patients remained in the progression-free health state as well as in the lifetime horizon longer than sorafenib patients, adding to the higher QALYs over a lifetime. The lower lenvatinib drug cost offset the higher medical resource cost incurred by lenvatinib patients who survived longer than sorafenib patients. Our results are robust to sensitivity analyses (DSA and PSA) and scenario analyses.

## Electronic supplementary material

Below is the link to the electronic supplementary material.

Supplementary material 1 (DOCX 54 kb)

## Footnotes

1 The exchange rate was $1 USD = 106.1575 JPY. Source http://www.exchangerate.com/. Site accessed March 7, 2018.
