# Supplementary material for: Cost-effectiveness analysis of lenvatinib treatment for patients with unresectable hepatocellular carcinoma (uHCC) compared with sorafenib in Japan
Source: J Gastroenterol. 2019 Feb 20;54(6):558–70. doi: 10.1007/s00535-019-01554-0 (PMC6536477; doi:10.1007/s00535-019-01554-0)
Supplement: Supplementary file 1 — Supplementary material 1 (DOCX 54 kb) [file 535_2019_1554_MOESM1_ESM.docx]

# Title

Cost-effectiveness analysis of lenvatinib treatment for patients with unresectable hepatocellular carcinoma (uHCC) compared with sorafenib in Japan.

# Authors

The full name(s) of the author(s)

Masahiro Kobayashi1), Masatoshi Kudo2), Namiki Izumi3), Shuichi Kaneko4),

Mie Azuma5), Ronda Copher6), Genevieve Meier 6), Janice Pan6), Mika Ishii5), Shunya Ikeda7)

The affiliation(s) and address(es) of the author(s)

1. Toranomon Hospital, Minato-ku, Tokyo, Japan
2. Kindai University, Osakasayama, Osaka, Japan
3. Musashino Red Cross Hospital, Musashino, Tokyo, Japan
4. Kanazawa University, Kanazawa, Ishikawa, Japan

5) Eisai Co., Ltd. Bunkyo-ku, Tokyo, Japan

6) Eisai Inc., Woodcliff Lake, NJ, USA

7) International University of Health and Welfare, Narita, Chiba, Japan

**• Short title (not exceeding 40 letters and spaces): 40**

Cost-effectiveness of lenvatinib in uHCC

# Electronic Supplemental Materials

#
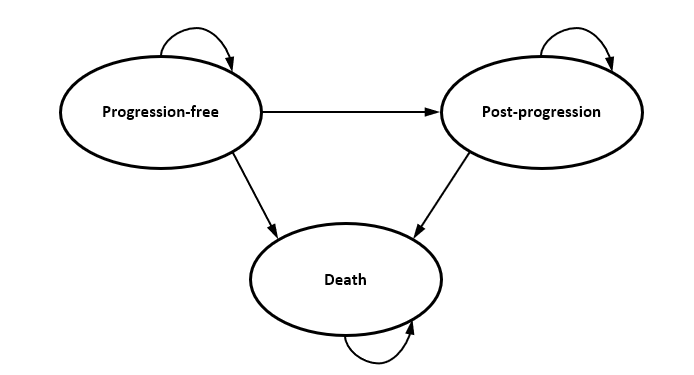


Supplemental figure 1. Model schematic for uHCC

The arrows represent hypothetical transitions between the health states. In a PSM approach, explicit movement between health states was not modelled, but instead, the distribution of patients across all health states at each cycle was modelled, defined by OS and PFS curves.

Supplemental table 1. Results of OS/PFS-ITT without stratification variables

| Treatment | Cost (JPY) | Effectiveness (LY) | Effectiveness (QALY) |
| --- | --- | --- | --- |
| Lenvatinib | 5 129 472 | 1.99 | 1.54 |
| Sorafenib | 5 573 617 | 1.84 | 1.39 |
| Incremental values | -444 145 | 0.15 | 0.15 |
| ICER |  | Dominant | Dominant |
